# Dicyclo­hexyl­ammonium trimethyl­bis­(hydrogen phenyl­phospho­nato)stannate(IV)

**DOI:** 10.1107/S1600536811049567

**Published:** 2011-11-30

**Authors:** Tidiane Diop, Libasse Diop, Cheikh A. K. Diop, Kieran C. Molloy, Gabriele Kociok-Köhn

**Affiliations:** a Laboratoire de Chimie Minérale et Analytique, Département de Chimie, Faculté des Sciences et Techniques, Université Cheikh Anta Diop, Dakar, Senegal; b Department of Chemistry, University of Bath, Claverton Down, Bath BA2 7AY, England

## Abstract

In the title compound, (C_12_H_24_N)[Sn(CH_3_)_3_(C_6_H_6_O_3_P)_2_], the SnMe_3_ residues are axially coordinated by two monodentate [PhPO_3_H]^−^ anions, leading to a trigonal–bipyramidal geometry for the Sn^IV^ atom. The two [SnMe_3_(PhPO_3_H)_2_]^−^ anions in the unit cell are associated into infinite chains along the *a* axis by O—H⋯O hydrogen bonds involving the hy­droxy group of the hydrogen phenyl­phospho­nate ion. The chains inter­act with one another *via* O—H⋯O hydrogen bonds along the *c* axis. These networks of anions assemble with the dicyclo­hexyl­ammonium ion through N—H⋯O hydrogen bonds, forming a three-dimensional network.

## Related literature

For related organotin derivatives, see: Weakley (1976 ▶); Molloy *et al.* (1981 ▶); Evans & Karpel (1985 ▶); Gielen *et al.* (1995 ▶); Yin & Wang (2004 ▶); Kapoor *et al.* (2005 ▶); Zhang *et al.* (2006 ▶). For our recent work on the coordination ability of oxyanions, see: Diop *et al.* (2002 ▶, 2003 ▶); Diallo *et al.* (2009 ▶).
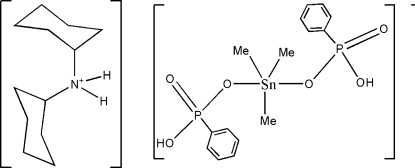


## Experimental

### 

#### Crystal data

(C_12_H_24_N)[Sn(CH_3_)_3_(C_6_H_6_O_3_P)_2_]
*M* *_r_* = 660.27
Triclinic, 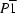

*a* = 10.8718 (5) Å
*b* = 12.7103 (7) Å
*c* = 13.3218 (7) Å
α = 100.625 (3)°
β = 103.687 (3)°
γ = 111.996 (3)°
*V* = 1580.41 (14) Å^3^
*Z* = 2
Mo *K*α radiation
μ = 0.95 mm^−1^
*T* = 150 K
0.45 × 0.30 × 0.20 mm

#### Data collection

Nonius KappaCCD diffractometer
Absorption correction: multi-scan (*SORTAV*; Blessing, 1995 ▶) *T*_min_ = 0.675, *T*_max_ = 0.833
21070 measured reflections
7212 independent reflections
6014 reflections with *I* > 2σ(*I*)
*R*_int_ = 0.068

#### Refinement

*R*[*F* ^2^ > 2σ(*F* ^2^)] = 0.047
*wR*(*F* ^2^) = 0.124
*S* = 1.09
7212 reflections
353 parameters
2 restraints
H atoms treated by a mixture of independent and constrained refinement
Δρ_max_ = 1.86 e Å^−3^
Δρ_min_ = −1.80 e Å^−3^

### 

Data collection: *COLLECT* (Nonius, 1998 ▶); cell refinement: *DENZO* and *SCALEPACK* (Otwinowski & Minor, 1997 ▶); data reduction: *DENZO* and *SCALEPACK* (Otwinowski & Minor, 1997 ▶); program(s) used to solve structure: *SIR97* (Altomare *et al.*, 1999 ▶); program(s) used to refine structure: *SHELXL97* (Sheldrick, 2008 ▶); molecular graphics: *ORTEP-3* (Farrugia, 1997 ▶); software used to prepare material for publication: *WinGX* (Farrugia, 1999 ▶).

## Supplementary Material

Crystal structure: contains datablock(s) I, global. DOI: 10.1107/S1600536811049567/vn2021sup1.cif

Structure factors: contains datablock(s) I. DOI: 10.1107/S1600536811049567/vn2021Isup2.hkl

Additional supplementary materials:  crystallographic information; 3D view; checkCIF report

## Figures and Tables

**Table 1:** Selected bond lengths (Å)

Sn—C1	2.132 (4)
Sn—C2	2.114 (4)
Sn—C3	2.134 (4)
Sn—O1	2.227 (2)
Sn—O4	2.241 (3)

**Table 2:** Hydrogen-bond geometry (Å, °)

*D*—H⋯*A*	*D*—H	H⋯*A*	*D*⋯*A*	*D*—H⋯*A*
O2—H2⋯O6^i^	0.76 (4)	1.88 (4)	2.642 (4)	178 (7)
O5—H5*A*⋯O6^ii^	0.94 (7)	1.67 (7)	2.596 (4)	169 (6)
N—H10*A*⋯O3	0.89 (2)	1.92 (2)	2.798 (4)	169 (4)
N—H10*B*⋯O3^iii^	0.95 (4)	1.83 (4)	2.759 (4)	165 (4)

Symmetry codes: (i) 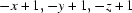; (ii) 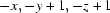; (iii) 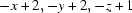.

**Fig. 1.**
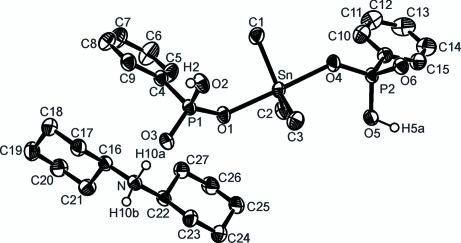
: The molecular structure of the title compound with the atom-numbering scheme. Displacement ellipsoids are drawn at the 50% probability level.

**Fig. 2.**
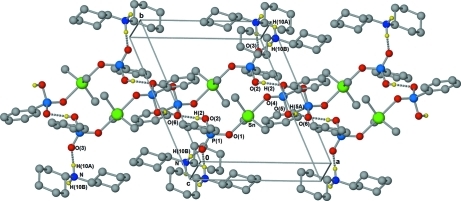
: Three dimensional structure showing the hydrogen bonds as dotted lines.

## supplementary crystallographic information

### Comment

Research on organotin derivatives has been an attractive area because of their numerous applications and their versatile structure (Evans & Karpel, 1985; Kapoor *et al.*, 2005; Zhang *et al.*, 2006; Yin & Wang, 2004; Gielen *et al.*, 1995). In the scope of our research work on the coordination ability of oxyanions (Diop *et al.*, 2002, Diallo *et al.*, 2009; Diop *et al.*, 2003) and our interest to synthesize new organotin derivatives for biological tests, we elucidate here the structure of the title compound, [C_15_H_21_O_6_P_2_Sn,C_12_H_24_N] (Fig. 1).

The crystal structure of the molecule is shown in Fig 2: hydrogen bonds between pairs of [SnMe_3_(PhPO_3_H)_2_]^-^ generate a six membered ring, comprise a honey comb network by virtue of the hydrogen bonds between the ligands as in *catena*-trimethyltin(IV)phenylarsenate, reported by Diop *et al. (*2002).

Each SnMe_3 _unit is σ bonded to two [PhPO_3_H]^-^ via one negatively charged oxygen atom, leading to a *trans* trigonal bipyramidal environment around the tin centre (Fig. 1). The resulting anions [SnMe_3_(PhPO_3_H)_2_]^- ^are associated through hydrogen bonds OH···O, along the *b* axis into pairs and along the *a* axis to form infinite layers (Fig. 2). The different layers are connected by NH···O hydrogen bonds along the *b* axis. The hydrogen bonds render the P=O and P—O bond distances almost egal (P(1)—O(3)1.506 (3) Å, P(1)—O(1): 1.509 (2) Å, P(1)—O(2): 1.569 (3) Å) while different of those in the parent phenylphosphonic acid PhPO_3_H_2_ (Weakley, 1976) (1.496 Å for P=O and 1.545 Å for P—OH). The geometry around the phosphorous atom in the ligands is a distorted tetrahedron (O(3)—P(1)—O(1): 115.48°(15), O(1)—P(1)—C(4): 107.03°(15)) owing to steric hindrance. The sum of the C—Sn—C angle is 59.99° and the O(1)—Sn—O(4) angle of 178.24°(9) indicate a nearly perfect *trans* trigonal bipyramidal arrangement with the carbon atoms of the methyl occupying the equatorial positions while the oxygen atoms are on the apical positions. The two Sn—O distances observed here - Sn—O(1) 2.227 (2) Å; Sn—O(4) 2.240 (3) Å - are shorter than the distances reported for(α-phenylphosphonato)trimethyltin(IV) by Molloy *et al.* (1981) (2.240 (6) Å and 2.319 (5) Å).

### Experimental

Cy_2_NH_2_PhPO_3_H (*L*) is obtained on mixing dicyclohexylamine with PhPO_3_H_2_ in water in 1/1 ratio. The title compound has been obtained as white crystalline solid by reacting (*L*) with trimetyltinchloride in ethanol (2/1 ratio *M*. p:170°). Slow solvent evaporation of the solution afforded colorless crystals suitable for x-ray structure determination. All the chemicals (Aldrich) were used without any further purification.

### Refinement

All C-bound H-atoms were positioned geometrically and were included in the refinement in the riding model approximation, with Uĩso~(H) set to 1.2 and 1.5 U~eq~(C). All N- and O-bound H-atoms have been located in the difference Fourier map and were refined freely. However, H(2) binding to O(2) and H(10A) binding to N had to be restrained with O—H = 0.82 (2) Å and N—H = 0.87 (2) Å.

### Crystal data

**Table:**

(C_12_H_24_N)[Sn(CH_3_)_3_(C_6_H_6_O_3_P)_2_]	*Z* = 2
*M**_r_* = 660.27	*F*(000) = 684
Triclinic, *P*1	*D*_x_ = 1.387 Mg m^−^^3^
Hall symbol: -P 1	Mo *K*α radiation, λ = 0.71073 Å
*a* = 10.8718 (5) Å	Cell parameters from 47204 reflections
*b* = 12.7103 (7) Å	θ = 2.9–27.5°
*c* = 13.3218 (7) Å	µ = 0.95 mm^−^^1^
α = 100.625 (3)°	*T* = 150 K
β = 103.687 (3)°	Plate, colourless
γ = 111.996 (3)°	0.45 × 0.30 × 0.20 mm
*V* = 1580.41 (14) Å^3^	

### Data collection

**Table:**

Nonius KappaCCD diffractometer	7212 independent reflections
Radiation source: fine-focus sealed tube	6014 reflections with *I* > 2σ(*I*)
graphite	*R*_int_ = 0.068
166 2.0 degree images with ω scans	θ_max_ = 27.5°, θ_min_ = 3.8°
Absorption correction: multi-scan (*SORTAV*; Blessing, 1995)	*h* = −14→14
*T*_min_ = 0.675, *T*_max_ = 0.833	*k* = −16→16
21070 measured reflections	*l* = −17→16

### Refinement

**Table:**

Refinement on *F*^2^	Primary atom site location: structure-invariant direct methods
Least-squares matrix: full	Secondary atom site location: difference Fourier map
*R*[*F*^2^ > 2σ(*F*^2^)] = 0.047	Hydrogen site location: inferred from neighbouring sites
*wR*(*F*^2^) = 0.124	H atoms treated by a mixture of independent and constrained refinement
*S* = 1.09	*w* = 1/[σ^2^(*F*_o_^2^) + (0.0655*P*)^2^ + 1.1841*P*] where *P* = (*F*_o_^2^ + 2*F*_c_^2^)/3
7212 reflections	(Δ/σ)_max_ < 0.001
353 parameters	Δρ_max_ = 1.86 e Å^−^^3^
2 restraints	Δρ_min_ = −1.80 e Å^−^^3^

### Special details

**Table:**

Geometry. All e.s.d.'s (except the e.s.d. in the dihedral angle between two l.s. planes) are estimated using the full covariance matrix. The cell e.s.d.'s are taken into account individually in the estimation of e.s.d.'s in distances, angles and torsion angles; correlations between e.s.d.'s in cell parameters are only used when they are defined by crystal symmetry. An approximate (isotropic) treatment of cell e.s.d.'s is used for estimating e.s.d.'s involving l.s. planes.
Refinement. Refinement of *F*^2^ against ALL reflections. The weighted *R*-factor *wR* and goodness of fit *S* are based on *F*^2^, conventional *R*-factors *R* are based on *F*, with *F* set to zero for negative *F*^2^. The threshold expression of *F*^2^ > σ(*F*^2^) is used only for calculating *R*-factors(gt) *etc*. and is not relevant to the choice of reflections for refinement. *R*-factors based on *F*^2^ are statistically about twice as large as those based on *F*, and *R*- factors based on ALL data will be even larger.

### Fractional atomic coordinates and isotropic or equivalent isotropic displacement parameters (Å^2^)

**Table:**

	*x*	*y*	*z*	*U*_iso_*/*U*_eq_	
Sn	0.39323 (2)	0.58948 (2)	0.341865 (19)	0.02776 (9)	
P1	0.74338 (9)	0.72317 (8)	0.37030 (7)	0.02711 (19)	
P2	0.06261 (9)	0.44906 (8)	0.36638 (8)	0.02827 (19)	
O1	0.6010 (2)	0.7236 (2)	0.3496 (2)	0.0320 (5)	
O2	0.7419 (3)	0.6228 (2)	0.4238 (2)	0.0353 (6)	
H2	0.807 (5)	0.623 (5)	0.462 (4)	0.070 (18)*	
O3	0.8675 (2)	0.8403 (2)	0.4370 (2)	0.0310 (5)	
O4	0.1834 (3)	0.4514 (2)	0.3290 (2)	0.0360 (6)	
O5	0.0892 (3)	0.5817 (2)	0.4150 (2)	0.0338 (6)	
H5A	0.039 (7)	0.585 (6)	0.463 (5)	0.08 (2)*	
O6	0.0351 (2)	0.3812 (2)	0.4473 (2)	0.0306 (5)	
N	0.9314 (3)	1.0380 (3)	0.3600 (2)	0.0291 (6)	
H10A	0.906 (5)	0.970 (3)	0.376 (4)	0.043 (12)*	
H10B	1.004 (4)	1.092 (4)	0.426 (3)	0.029 (10)*	
C1	0.4197 (4)	0.4478 (4)	0.2508 (3)	0.0357 (8)	
H1A	0.3304	0.3754	0.2223	0.053*	
H1B	0.4921	0.4334	0.2977	0.053*	
H1C	0.4488	0.4694	0.1904	0.053*	
C2	0.4641 (4)	0.6421 (4)	0.5132 (3)	0.0373 (8)	
H2A	0.5502	0.6326	0.5399	0.056*	
H2B	0.3912	0.5924	0.5378	0.056*	
H2C	0.4837	0.7257	0.5412	0.056*	
C3	0.2929 (4)	0.6733 (4)	0.2497 (3)	0.0396 (9)	
H3A	0.2375	0.6994	0.2876	0.059*	
H3B	0.2307	0.6166	0.1782	0.059*	
H3C	0.3646	0.7424	0.2409	0.059*	
C4	0.7614 (4)	0.6798 (3)	0.2399 (3)	0.0297 (7)	
C5	0.6643 (4)	0.6721 (4)	0.1461 (3)	0.0433 (10)	
H5	0.5856	0.6861	0.1506	0.052*	
C6	0.6811 (6)	0.6443 (6)	0.0462 (4)	0.0610 (14)	
H6	0.6148	0.6405	−0.0170	0.073*	
C7	0.7947 (5)	0.6219 (5)	0.0383 (3)	0.0516 (11)	
H7	0.8058	0.6026	−0.0304	0.062*	
C8	0.8919 (5)	0.6278 (4)	0.1304 (4)	0.0430 (9)	
H8	0.9689	0.6115	0.1252	0.052*	
C9	0.8754 (4)	0.6579 (3)	0.2309 (3)	0.0355 (8)	
H9	0.9430	0.6635	0.2942	0.043*	
C10	−0.0932 (4)	0.3826 (3)	0.2475 (3)	0.0322 (7)	
C11	−0.0850 (5)	0.3461 (4)	0.1446 (3)	0.0444 (10)	
H11	0.0032	0.3562	0.1372	0.053*	
C12	−0.2058 (6)	0.2946 (5)	0.0527 (4)	0.0562 (12)	
H12	−0.2001	0.2691	−0.0170	0.067*	
C13	−0.3337 (6)	0.2812 (5)	0.0639 (4)	0.0595 (13)	
H13	−0.4159	0.2462	0.0014	0.071*	
C14	−0.3430 (5)	0.3180 (4)	0.1645 (4)	0.0512 (11)	
H14	−0.4314	0.3085	0.1711	0.061*	
C15	−0.2241 (4)	0.3688 (4)	0.2559 (3)	0.0391 (9)	
H15	−0.2312	0.3946	0.3250	0.047*	
C16	0.9824 (4)	1.0236 (3)	0.2651 (3)	0.0314 (7)	
H16	0.9017	0.9615	0.2010	0.038*	
C17	1.0365 (5)	1.1398 (4)	0.2371 (3)	0.0406 (9)	
H17A	1.1137	1.2033	0.3008	0.049*	
H17B	0.9597	1.1641	0.2179	0.049*	
C18	1.0900 (5)	1.1238 (4)	0.1419 (4)	0.0465 (10)	
H18A	1.1312	1.2012	0.1279	0.056*	
H18B	1.0099	1.0674	0.0761	0.056*	
C19	1.2011 (5)	1.0767 (4)	0.1639 (4)	0.0470 (10)	
H19A	1.2276	1.0617	0.0984	0.056*	
H19B	1.2864	1.1377	0.2237	0.056*	
C20	1.1468 (4)	0.9626 (4)	0.1941 (4)	0.0438 (9)	
H20A	1.2227	0.9371	0.2122	0.053*	
H20B	1.0678	0.8990	0.1315	0.053*	
C21	1.0967 (4)	0.9810 (4)	0.2915 (3)	0.0376 (8)	
H21A	1.0590	0.9053	0.3089	0.045*	
H21B	1.1770	1.0408	0.3557	0.045*	
C22	0.8128 (4)	1.0751 (3)	0.3484 (3)	0.0325 (8)	
H22	0.8446	1.1542	0.3349	0.039*	
C23	0.7833 (4)	1.0885 (4)	0.4551 (3)	0.0395 (9)	
H23A	0.7567	1.0119	0.4717	0.047*	
H23B	0.8695	1.1491	0.5144	0.047*	
C24	0.6648 (4)	1.1259 (4)	0.4481 (4)	0.0472 (10)	
H24A	0.6941	1.2052	0.4367	0.057*	
H24B	0.6447	1.1319	0.5171	0.057*	
C25	0.5317 (4)	1.0355 (4)	0.3547 (4)	0.0499 (11)	
H25A	0.4982	0.9577	0.3690	0.060*	
H25B	0.4568	1.0626	0.3497	0.060*	
C26	0.5605 (4)	1.0211 (4)	0.2482 (4)	0.0493 (11)	
H26A	0.5843	1.0969	0.2302	0.059*	
H26B	0.4745	0.9591	0.1897	0.059*	
C27	0.6824 (4)	0.9860 (4)	0.2541 (3)	0.0377 (8)	
H27A	0.6544	0.9057	0.2632	0.045*	
H27B	0.7035	0.9833	0.1856	0.045*	

### Atomic displacement parameters (Å^2^)

**Table:**

	*U*^11^	*U*^22^	*U*^33^	*U*^12^	*U*^13^	*U*^23^
Sn	0.02108 (13)	0.03255 (14)	0.03159 (14)	0.01278 (10)	0.00933 (9)	0.01115 (9)
P1	0.0195 (4)	0.0280 (4)	0.0322 (4)	0.0092 (4)	0.0066 (3)	0.0106 (3)
P2	0.0206 (4)	0.0297 (5)	0.0366 (5)	0.0116 (4)	0.0107 (3)	0.0121 (4)
O1	0.0219 (12)	0.0349 (13)	0.0415 (14)	0.0131 (11)	0.0104 (10)	0.0156 (11)
O2	0.0234 (13)	0.0372 (14)	0.0439 (15)	0.0105 (11)	0.0067 (11)	0.0213 (12)
O3	0.0209 (11)	0.0320 (13)	0.0333 (13)	0.0073 (10)	0.0059 (9)	0.0086 (10)
O4	0.0254 (12)	0.0364 (14)	0.0504 (16)	0.0141 (11)	0.0181 (11)	0.0146 (12)
O5	0.0296 (13)	0.0310 (13)	0.0426 (14)	0.0128 (11)	0.0144 (11)	0.0134 (11)
O6	0.0243 (12)	0.0317 (13)	0.0379 (13)	0.0132 (11)	0.0100 (10)	0.0138 (10)
N	0.0240 (14)	0.0287 (16)	0.0314 (15)	0.0098 (13)	0.0065 (12)	0.0097 (12)
C1	0.0299 (18)	0.041 (2)	0.039 (2)	0.0182 (17)	0.0129 (15)	0.0108 (16)
C2	0.038 (2)	0.045 (2)	0.038 (2)	0.0237 (18)	0.0148 (16)	0.0166 (17)
C3	0.0293 (18)	0.045 (2)	0.048 (2)	0.0175 (17)	0.0108 (16)	0.0216 (18)
C4	0.0246 (16)	0.0316 (18)	0.0337 (18)	0.0129 (15)	0.0089 (13)	0.0116 (14)
C5	0.034 (2)	0.061 (3)	0.037 (2)	0.028 (2)	0.0082 (16)	0.0097 (18)
C6	0.054 (3)	0.100 (4)	0.036 (2)	0.048 (3)	0.008 (2)	0.013 (2)
C7	0.057 (3)	0.074 (3)	0.034 (2)	0.036 (3)	0.0198 (19)	0.014 (2)
C8	0.042 (2)	0.049 (2)	0.049 (2)	0.029 (2)	0.0184 (18)	0.0150 (19)
C9	0.0273 (18)	0.039 (2)	0.041 (2)	0.0154 (16)	0.0094 (15)	0.0140 (16)
C10	0.0314 (18)	0.0330 (19)	0.0383 (19)	0.0183 (16)	0.0124 (15)	0.0146 (15)
C11	0.047 (2)	0.054 (3)	0.042 (2)	0.029 (2)	0.0163 (18)	0.0181 (19)
C12	0.064 (3)	0.067 (3)	0.039 (2)	0.037 (3)	0.009 (2)	0.014 (2)
C13	0.053 (3)	0.059 (3)	0.050 (3)	0.030 (3)	−0.009 (2)	0.005 (2)
C14	0.031 (2)	0.051 (3)	0.063 (3)	0.021 (2)	0.0031 (19)	0.009 (2)
C15	0.0289 (18)	0.042 (2)	0.045 (2)	0.0182 (17)	0.0095 (16)	0.0103 (17)
C16	0.0270 (17)	0.0316 (18)	0.0313 (17)	0.0097 (15)	0.0087 (14)	0.0080 (14)
C17	0.047 (2)	0.036 (2)	0.044 (2)	0.0195 (19)	0.0187 (18)	0.0167 (17)
C18	0.055 (3)	0.046 (2)	0.044 (2)	0.021 (2)	0.025 (2)	0.0191 (19)
C19	0.039 (2)	0.052 (3)	0.049 (2)	0.016 (2)	0.0211 (19)	0.015 (2)
C20	0.035 (2)	0.046 (2)	0.050 (2)	0.0188 (19)	0.0157 (18)	0.0097 (19)
C21	0.0325 (19)	0.038 (2)	0.043 (2)	0.0164 (17)	0.0136 (16)	0.0127 (16)
C22	0.0248 (17)	0.0293 (18)	0.042 (2)	0.0122 (15)	0.0079 (14)	0.0116 (15)
C23	0.0301 (19)	0.044 (2)	0.042 (2)	0.0158 (17)	0.0132 (16)	0.0075 (17)
C24	0.033 (2)	0.047 (2)	0.058 (3)	0.0178 (19)	0.0167 (19)	0.006 (2)
C25	0.0269 (19)	0.044 (2)	0.071 (3)	0.0136 (18)	0.0155 (19)	0.005 (2)
C26	0.029 (2)	0.049 (3)	0.058 (3)	0.0165 (19)	0.0013 (18)	0.008 (2)
C27	0.0280 (18)	0.041 (2)	0.040 (2)	0.0156 (17)	0.0054 (15)	0.0096 (16)

### Geometric parameters (Å, °)

**Table:**

Sn—C1	2.132 (4)	C11—H11	0.9500
Sn—C2	2.114 (4)	C12—C13	1.383 (8)
Sn—C3	2.134 (4)	C12—H12	0.9500
Sn—O1	2.227 (2)	C13—C14	1.376 (7)
Sn—O4	2.241 (3)	C13—H13	0.9500
P1—O3	1.506 (3)	C14—C15	1.383 (6)
P1—O1	1.509 (2)	C14—H14	0.9500
P1—O2	1.569 (3)	C15—H15	0.9500
P1—C4	1.803 (4)	C16—C17	1.524 (5)
P2—O4	1.503 (3)	C16—C21	1.525 (5)
P2—O6	1.516 (3)	C16—H16	1.0000
P2—O5	1.578 (3)	C17—C18	1.527 (6)
P2—C10	1.805 (4)	C17—H17A	0.9900
O2—H2	0.76 (4)	C17—H17B	0.9900
O5—H5A	0.94 (7)	C18—C19	1.529 (6)
N—C16	1.503 (5)	C18—H18A	0.9900
N—C22	1.515 (5)	C18—H18B	0.9900
N—H10A	0.887 (19)	C19—C20	1.517 (6)
N—H10B	0.95 (4)	C19—H19A	0.9900
C1—H1A	0.9800	C19—H19B	0.9900
C1—H1B	0.9800	C20—C21	1.534 (6)
C1—H1C	0.9800	C20—H20A	0.9900
C2—H2A	0.9800	C20—H20B	0.9900
C2—H2B	0.9800	C21—H21A	0.9900
C2—H2C	0.9800	C21—H21B	0.9900
C3—H3A	0.9800	C22—C27	1.517 (5)
C3—H3B	0.9800	C22—C23	1.522 (5)
C3—H3C	0.9800	C22—H22	1.0000
C4—C5	1.393 (5)	C23—C24	1.521 (5)
C4—C9	1.395 (5)	C23—H23A	0.9900
C5—C6	1.385 (6)	C23—H23B	0.9900
C5—H5	0.9500	C24—C25	1.528 (6)
C6—C7	1.390 (7)	C24—H24A	0.9900
C6—H6	0.9500	C24—H24B	0.9900
C7—C8	1.386 (6)	C25—C26	1.517 (7)
C7—H7	0.9500	C25—H25A	0.9900
C8—C9	1.395 (6)	C25—H25B	0.9900
C8—H8	0.9500	C26—C27	1.539 (6)
C9—H9	0.9500	C26—H26A	0.9900
C10—C11	1.399 (5)	C26—H26B	0.9900
C10—C15	1.401 (5)	C27—H27A	0.9900
C11—C12	1.398 (6)	C27—H27B	0.9900
			
C2—Sn—C1	121.15 (15)	C12—C13—H13	119.7
C2—Sn—C3	122.89 (16)	C13—C14—C15	120.1 (4)
C1—Sn—C3	115.97 (16)	C13—C14—H14	119.9
C2—Sn—O1	88.92 (13)	C15—C14—H14	119.9
C1—Sn—O1	91.77 (12)	C14—C15—C10	120.6 (4)
C3—Sn—O1	89.14 (13)	C14—C15—H15	119.7
C2—Sn—O4	92.68 (13)	C10—C15—H15	119.7
C1—Sn—O4	86.78 (13)	N—C16—C17	110.9 (3)
C3—Sn—O4	90.59 (13)	N—C16—C21	109.1 (3)
O1—Sn—O4	178.24 (9)	C17—C16—C21	111.2 (3)
O3—P1—O1	115.48 (15)	N—C16—H16	108.5
O3—P1—O2	110.97 (15)	C17—C16—H16	108.5
O1—P1—O2	107.81 (15)	C21—C16—H16	108.5
O3—P1—C4	108.23 (15)	C16—C17—C18	110.1 (3)
O1—P1—C4	107.03 (15)	C16—C17—H17A	109.6
O2—P1—C4	106.94 (16)	C18—C17—H17A	109.6
O4—P2—O6	115.57 (15)	C16—C17—H17B	109.6
O4—P2—O5	108.23 (15)	C18—C17—H17B	109.6
O6—P2—O5	110.03 (15)	H17A—C17—H17B	108.2
O4—P2—C10	107.22 (17)	C17—C18—C19	111.8 (4)
O6—P2—C10	108.73 (16)	C17—C18—H18A	109.3
O5—P2—C10	106.67 (16)	C19—C18—H18A	109.3
P1—O1—Sn	132.28 (15)	C17—C18—H18B	109.3
P1—O2—H2	125 (5)	C19—C18—H18B	109.3
P2—O4—Sn	136.69 (16)	H18A—C18—H18B	107.9
P2—O5—H5A	110 (4)	C20—C19—C18	111.4 (4)
C16—N—C22	117.5 (3)	C20—C19—H19A	109.3
C16—N—H10A	107 (3)	C18—C19—H19A	109.3
C22—N—H10A	109 (3)	C20—C19—H19B	109.3
C16—N—H10B	114 (2)	C18—C19—H19B	109.3
C22—N—H10B	106 (2)	H19A—C19—H19B	108.0
H10A—N—H10B	102 (4)	C19—C20—C21	110.7 (3)
Sn—C1—H1A	109.5	C19—C20—H20A	109.5
Sn—C1—H1B	109.5	C21—C20—H20A	109.5
H1A—C1—H1B	109.5	C19—C20—H20B	109.5
Sn—C1—H1C	109.5	C21—C20—H20B	109.5
H1A—C1—H1C	109.5	H20A—C20—H20B	108.1
H1B—C1—H1C	109.5	C16—C21—C20	109.7 (3)
Sn—C2—H2A	109.5	C16—C21—H21A	109.7
Sn—C2—H2B	109.5	C20—C21—H21A	109.7
H2A—C2—H2B	109.5	C16—C21—H21B	109.7
Sn—C2—H2C	109.5	C20—C21—H21B	109.7
H2A—C2—H2C	109.5	H21A—C21—H21B	108.2
H2B—C2—H2C	109.5	N—C22—C27	111.3 (3)
Sn—C3—H3A	109.5	N—C22—C23	108.1 (3)
Sn—C3—H3B	109.5	C27—C22—C23	111.6 (3)
H3A—C3—H3B	109.5	N—C22—H22	108.6
Sn—C3—H3C	109.5	C27—C22—H22	108.6
H3A—C3—H3C	109.5	C23—C22—H22	108.6
H3B—C3—H3C	109.5	C24—C23—C22	110.4 (3)
C5—C4—C9	118.5 (3)	C24—C23—H23A	109.6
C5—C4—P1	120.5 (3)	C22—C23—H23A	109.6
C9—C4—P1	121.0 (3)	C24—C23—H23B	109.6
C6—C5—C4	120.8 (4)	C22—C23—H23B	109.6
C6—C5—H5	119.6	H23A—C23—H23B	108.1
C4—C5—H5	119.6	C23—C24—C25	110.5 (4)
C5—C6—C7	120.1 (4)	C23—C24—H24A	109.5
C5—C6—H6	119.9	C25—C24—H24A	109.5
C7—C6—H6	119.9	C23—C24—H24B	109.5
C8—C7—C6	120.1 (4)	C25—C24—H24B	109.5
C8—C7—H7	120.0	H24A—C24—H24B	108.1
C6—C7—H7	120.0	C26—C25—C24	110.8 (4)
C7—C8—C9	119.4 (4)	C26—C25—H25A	109.5
C7—C8—H8	120.3	C24—C25—H25A	109.5
C9—C8—H8	120.3	C26—C25—H25B	109.5
C8—C9—C4	121.1 (4)	C24—C25—H25B	109.5
C8—C9—H9	119.4	H25A—C25—H25B	108.1
C4—C9—H9	119.4	C25—C26—C27	111.4 (4)
C11—C10—C15	118.6 (4)	C25—C26—H26A	109.3
C11—C10—P2	120.5 (3)	C27—C26—H26A	109.3
C15—C10—P2	120.9 (3)	C25—C26—H26B	109.3
C12—C11—C10	120.3 (4)	C27—C26—H26B	109.3
C12—C11—H11	119.8	H26A—C26—H26B	108.0
C10—C11—H11	119.8	C22—C27—C26	110.4 (3)
C13—C12—C11	119.6 (5)	C22—C27—H27A	109.6
C13—C12—H12	120.2	C26—C27—H27A	109.6
C11—C12—H12	120.2	C22—C27—H27B	109.6
C14—C13—C12	120.7 (4)	C26—C27—H27B	109.6
C14—C13—H13	119.7	H27A—C27—H27B	108.1

### Hydrogen-bond geometry (Å, °)

**Table:**

*D*—H···*A*	*D*—H	H···*A*	*D*···*A*	*D*—H···*A*
O2—H2···O6^i^	0.76 (4)	1.88 (4)	2.642 (4)	178 (7)
O5—H5A···O6^ii^	0.94 (7)	1.67 (7)	2.596 (4)	169 (6)
N—H10A···O3	0.89 (2)	1.92 (2)	2.798 (4)	169 (4)
N—H10B···O3^iii^	0.95 (4)	1.83 (4)	2.759 (4)	165 (4)

Symmetry codes: (i) −*x*+1, −*y*+1, −*z*+1; (ii) −*x*, −*y*+1, −*z*+1; (iii) −*x*+2, −*y*+2, −*z*+1.
